# Supplementary material for: Chromothripsis during telomere crisis is independent of NHEJ, and consistent with a replicative origin
Source: Genome Res. 2019 May;29(5):737–49. doi: 10.1101/gr.240705.118 (PMC6499312; doi:10.1101/gr.240705.118)
Supplement: Supplemental Material [file supp_gr.240705.118_Supplemental_file_1.zip › contigs/annotated_contigs/DB50/contig.3.DB50_length_546_mean_cov_4.93223443223.docx]

**DB50_length_546_mean_cov_4.93223443223**

```
TCACCACTCCTCAAACATCCCTTAACATCGCAGAAATTTAACCAGCTCTGTTAAAAGTGTATATTTTAGACTGGAATATATATTTTGCC
 >chr3:177740826-177741060 - E=2e-124
CCTCAAGTTATAATAATGTATTTGAGATTCAAAAGAAATAAAAGTGGATTGCACAAAATACAGCTCATTTCCCCAGAAGGTAAATGTTA

AGGGATTTTTTTTTAAGCAGACCAAGTGACTTCTTTCATTTTATATATATATATA|AAATG|AAACTATCAAATTGCGCATTTCAAATG
                                                             >chr10:36330477-36330520 -
GGTGAATTTTGTGGTA|AAATG|GCTATTGTAAGGAATGAAGATAGTGGGATTTTGACAGTTTCCTGATCCTGCTTTATTTTTTCCCCA
 E=3e-14              >chr10:36439438-36439698 + E=8e-145
AAATGTTTCTGTATCTGAAAAACTGAAACTATAGTCATCCTAAACTTCTGATTCTGTAAGCAGAAGAAACAGCAACGGCTTGAATTTTT

ACTTCCTTCTAACATTTTCTATTTATGATTTAATATGAGTGATTATTCTAATATTTTAGTGTAGGTCATTATCACTTGGTGGTATTATT

AAAAATACAAATGCCT
```
